# Delivering emergency care at the religious mass gathering of India: an amalgamation of technology and spirituality—an institutional experience of managing pilgrims at Mahakumbh Prayagraj

**DOI:** 10.3389/fpubh.2025.1695241

**Published:** 2025-12-17

**Authors:** Suyash Singh, Kumari Shaloo, Shruti Sinha, Purushottam Kumar, Bhola Nath

**Affiliations:** ^1^Department of Neurosurgery, All India Institute of Medical Sciences Raebareli, Raebareli, India; ^2^Department of Psychiatry, All India Institute of Medical Sciences Raebareli, Raebareli, India; ^3^Department of Community and Family Medicine, All India Institute of Medical Sciences Raebareli, Raebareli, India

**Keywords:** Mahakumbh, religious mass gathering, public health preparedness, endemicity, technology-enabled healthcare systems, respiratory tract infections, myocardial infarction, artificial intelligence

## Abstract

**Background:**

The Mahakumbh Mela 2025 in Prayagraj, India, was one of the world’s largest religious mass gatherings, hosting more than 660 million pilgrims. Delivering effective emergency and inpatient care in this dynamic, resource-limited setting posed unique challenges, including risk of infectious disease outbreaks, environmental stressors, and infrastructural constraints. This study documents the institutional experience of managing healthcare services during the event.

**Methodology:**

This retrospective, mixed-methods study was conducted at the Sub-Center Hospital Sector 20 organised by AIIMS, Raebareli (UP). All patients attending between 8 January and 24 February 2025 were included. Quantitative data from 57,950 OPD and 1,190 IPD records including electronic medical records, laboratory reports, and field journals were analysed using SPSS v24. Qualitative insights were obtained from reflective journals and operational notes maintained by healthcare personnel. These were thematically summarised through an inductive, pragmatic approach to identify contextual challenges, workflow dynamics, and adaptive strategies. Ethical approval (IEC No. 2024-5-OTH-EXP-9) and administrative permissions were obtained.

**Discussion:**

Fever (30.6%), respiratory (23.8%), and gastrointestinal (22.0%) illnesses predominated in OPD cases. IPD admissions most frequently involved SOB (37.6%), chest pain (4.4%), and seizures (1.6%), with 3.2% acute myocardial infarctions identified. Key challenges included crowd density, dust exposure, and limited access to care. Skill-building trained over 1,000 personnel in emergency protocols and BLS, contributing to effective healthcare delivery.

**Conclusion:**

This institutional experience underscores the critical need for robust preparedness, skilled manpower, advanced diagnostics, and technology enabled coordination in managing healthcare during mass gatherings. The success at Mahakumbh 2025 demonstrates that large-scale health crises can be effectively mitigated with multidisciplinary coordination and strategic preparedness.

## Introduction

Organising a health facility for more than 60 million pilgrims and that too along the banks of river Ganga, was not only a challenging task but also required a lot of planning. An ‘emergency response’ facility, that too in a place where oxygen inflow had to be planned on temporary basis, and all ventilators had to be managed on a rotation shift, was a difficult task. The Mahakumbh Mela, a massive religious gathering in India, presents unique challenges to healthcare providers due to the sheer scale of the event and the potential for mass casualty incidents. Providing medical care at the Mahakumbh Mela requires a comprehensive understanding of disaster medicine principles, infectious disease management, and logistical coordination. Globally, mass gatherings such as the Hajj, the Olympics, and large humanitarian missions have fostered the discipline of mass-gathering medicine, which advances planning, surveillance, and intersectoral coordination in high-density, culturally diverse settings. The Mahakumbh represents a related but unique Indian model wherein faith-driven participation interacts with modern public health strategies and technology-enabled healthcare delivery. This article depicts our institutional experience of planning, managing and treating thousands of patients in our “Sub-Center Hospital Sector 20 Mahakumbh” organised by AIIMS, Raebareli (UP). This is one of the first studies to systematically describe an integrated framework for emergency and inpatient healthcare during the Mahakumbh Mela 2025, demonstrating how digital health systems, modern medical technology, and faith-based motivation synergistically enabled efficient triage, rapid clinical decision-making, and culturally sensitive care in a high-density, resource-constrained setting. We managed the OPDs, IPDs and ICUs with 24-h emergency services. The facility was equipped with all necessary equipments and medicines along with jumbo oxygen cylinders, being transported from our main institute hospital (more than a 100 km distant). Our experience of managing emergency services and ICU care during the Mahakumbh Mela will provide valuable insights into the complexities of delivering healthcare in such an extraordinary setting. The convergence of millions of pilgrims creates a heightened risk of outbreaks, injuries, and other health emergencies, demanding a robust and adaptable medical response. The psychological effect of COVID-19 pandemic had further complicated mass gatherings like the Kumbh Mela, as these events can amplify the spread of infectious diseases. Effective crowd management strategies are essential to mitigating risks and ensuring the safety of attendees at mass gatherings like the Kumbh Mela. The lack of such measures, along with ‘temporary’ infrastructure, has been implicated in mismanagements in previous Kumbh Mela events. The challenges are compounded by the potential for a surge in patients with chronic diseases whose conditions may be exacerbated by the arduous conditions of travel and the environment of the Mela.

**Figure 1:**
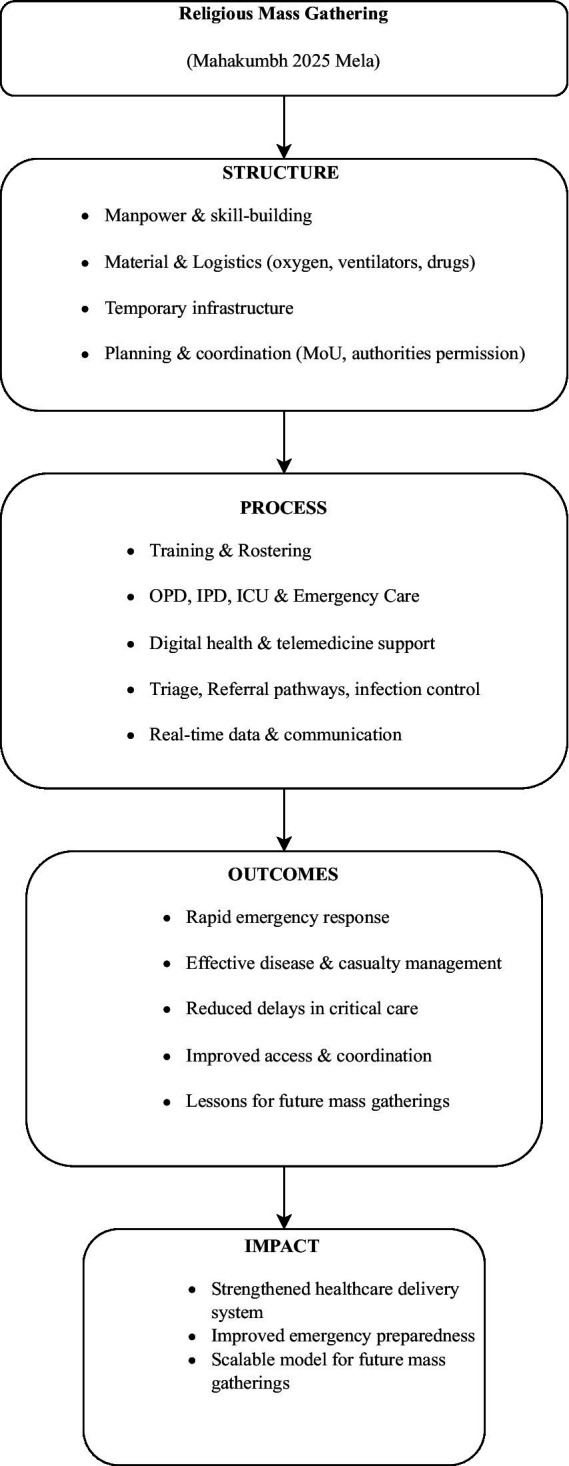
Conceptual framework illustrating the integrated, technology-enabled emergency healthcare response model during the Mahakumbh Mela 2025, Prayagraj—adapted from Donabedian’s Structure–Process–Outcome model and the WHO Health Systems Framework to demonstrate linkages between infrastructure, service delivery, and health outcomes.

Our Sub-Center hospital served pilgrims from 8th January 2025 to 24th February 2025 and we present our data of these 45-days. In this paper, we will be discussing and focusing on the following aspects of managing the hospital. Conceptual framework (Figure 1), developed form the Donabedian (Structure-Process-Outcome model) and WHO (Health Systems Framework), depicting the technology-enabled emergency healthcare response utilised during the Mahakumbh Mela 2025 in Prayagraj (1).

**Figure 2:**
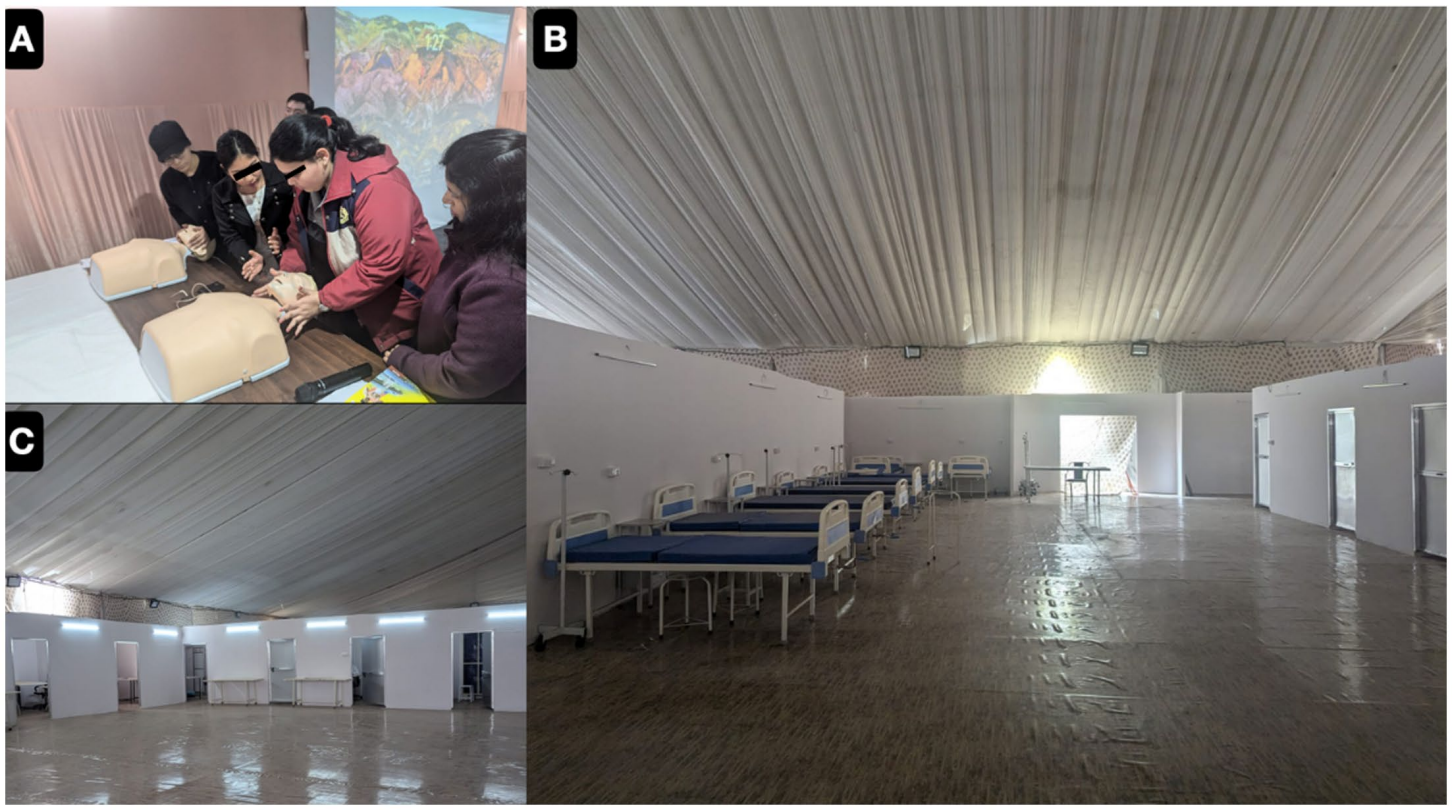
Our commitment started with training phase which comprises a 5-days programme on basic life support and disaster preparedness training in coordination with Uttar Pradesh Medical Health Department **(A)**. Around last week of December 2024, our temporary hospital was ready with basic infrastructures and hospital furniture’s **(B,C)**.

- Planning phase (manpower, material and logistics) as illustrated in Figure 2.
- Execution (trainings, rosters and clinical management)
- OPD management
- ICU management
- Challenges in logistics and local issues
- Strategies for similar future “Religious Mass Gatherings”

## Methodology

### Study design and setting

**Figure 3:**
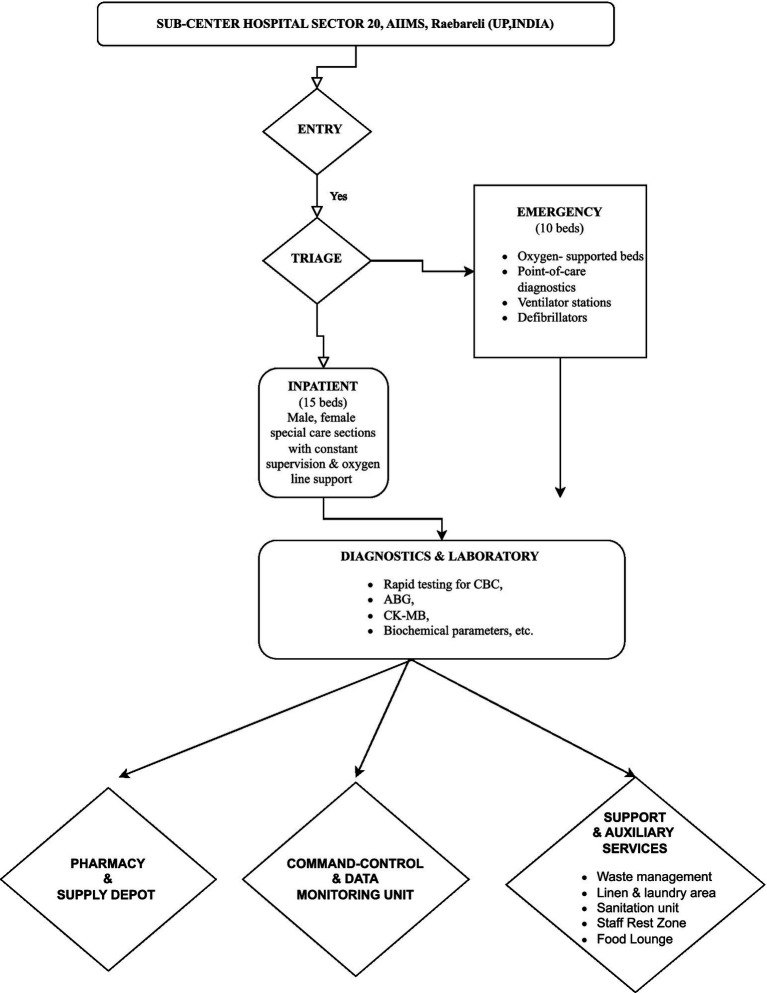
Schematic layout of the SUB-CENTER HOSPITAL AIIMS Mahakumbh, depicting the structured patient flowfrom entry and triage to emergency, inpatient, diagnostic, and support zones established during the Mahakumbh–2025 event.

This retrospective mixed-methods study was conducted at the Sub-Center Hospital, Sector-20, established by the All India Institute of Medical Sciences (AIIMS) Raebareli during the Mahakumbh Mela 2025 in Prayagraj. The study focused on capturing the nuances of emergency care delivery during a high-density religious mass gathering. It draws on the direct clinical experience of a physician actively involved in healthcare services to pilgrims throughout the event as shown in Figure 3.

### Study participants and sampling

All patients who sought treatment at the Sub-Center Hospital between January 8 and February 24, 2025 were included. The dataset included OPD visits, emergency cases, and IPD admissions. No sampling was performed since all consecutive cases recorded during the study period were retrospectively analysed.

### Data collection tool and technique

Quantitative data were obtained from electronically maintained OPD records, IPD case files, laboratory reports, and hospital registries, capturing demographics, presenting complaints, diagnoses, treatments, and outcomes. Qualitative insights were derived from reflective field journals and operational notes maintained by healthcare personnel. These were thematically analysed through an inductive, pragmatic approach to identify operational challenges, workflow dynamics, and adaptive strategies.

### Ethical consideration

The study received approval from the Institutional Ethics Committee (IEC No. 2024-5-OTH-EXP-9). Administrative permissions were obtained through a Memorandum of Understanding (MoU) with local authorities. As anonymised institutional records were used, informed consent was not required.

### Statistical analysis method

Data were analysed using SPSS version 24.0. Descriptive statistics were used to summarise quantitative variables, and a *p*-value <0.05 was considered statistically significant. Qualitative findings were thematically analysed and triangulated with quantitative trends to enhance interpretive reliability.

## Results

### Epidemiological distribution and clinical presentation: outpatient department data analyses

**Figure 4:**
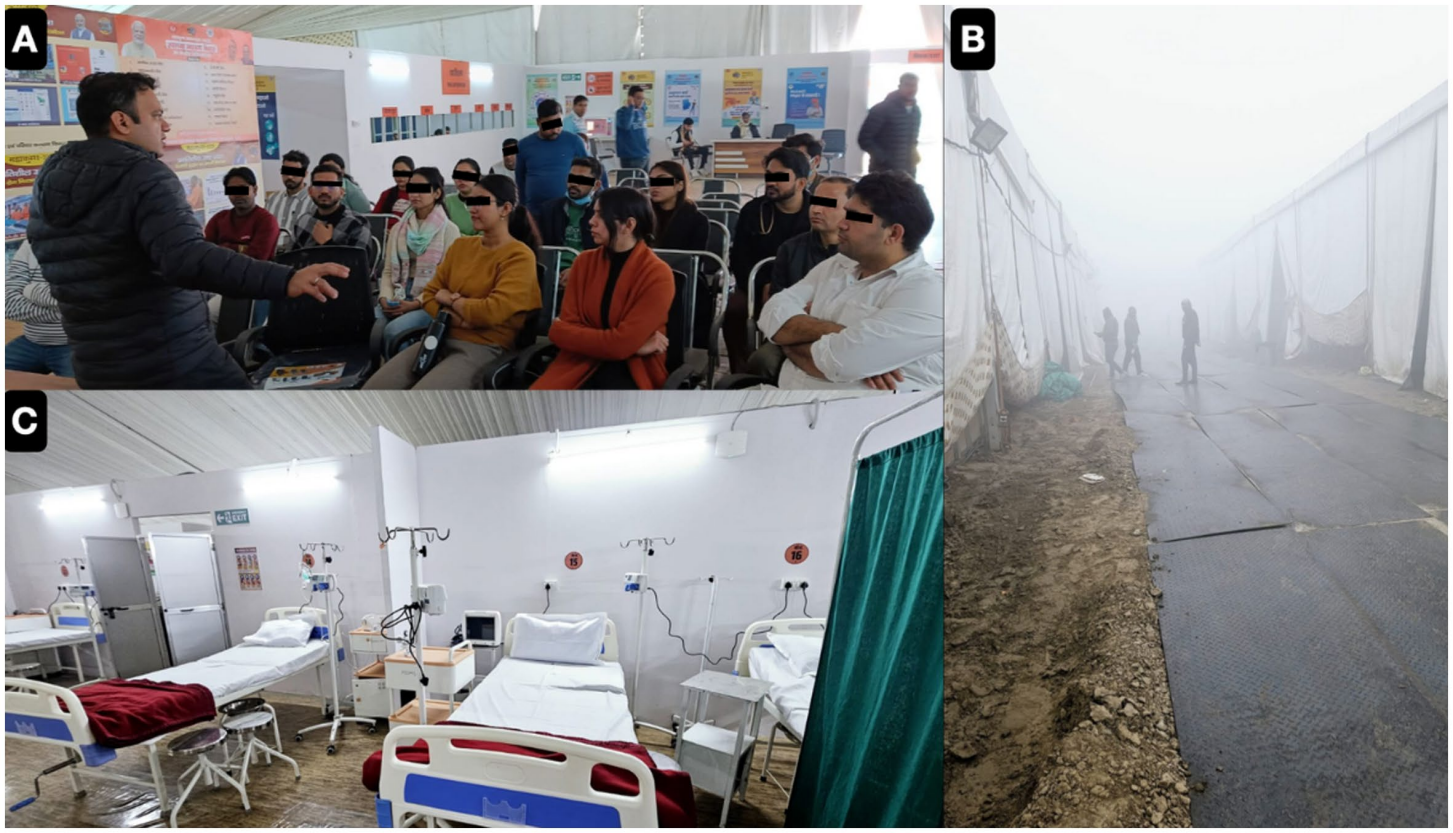
The key to successful organisation of outreach activity in harsh climate and crowded moving patient population was briefing and role assignment with upgrading the basic clinical skills in each cadre **(A)**. Self motivation and inclination of each health care staff towards religious belief and wish to work in religious gathering kept us going even in extreme cold winters **(B)**. Around first week of January, our ICU was set-up after transporting necessary gadgets from our mother institute **(C)**.

During the sacred observance of Mahakumbh 2025, held from January 9 to February 27 in Prayagraj, a retrospective epidemiological audit was conducted by both active and passive surveillance from medical outreach camps. According to remotely recorded OPD data from attendees of the massive crowd. A total of 57,950 individuals were diagnosed with various clinical conditions, reflecting a high burden of symptoms among those seeking care by the healthcare team Figure 4 representing OPD services as serving the pilgrims across the globe.

**Figure 5:**
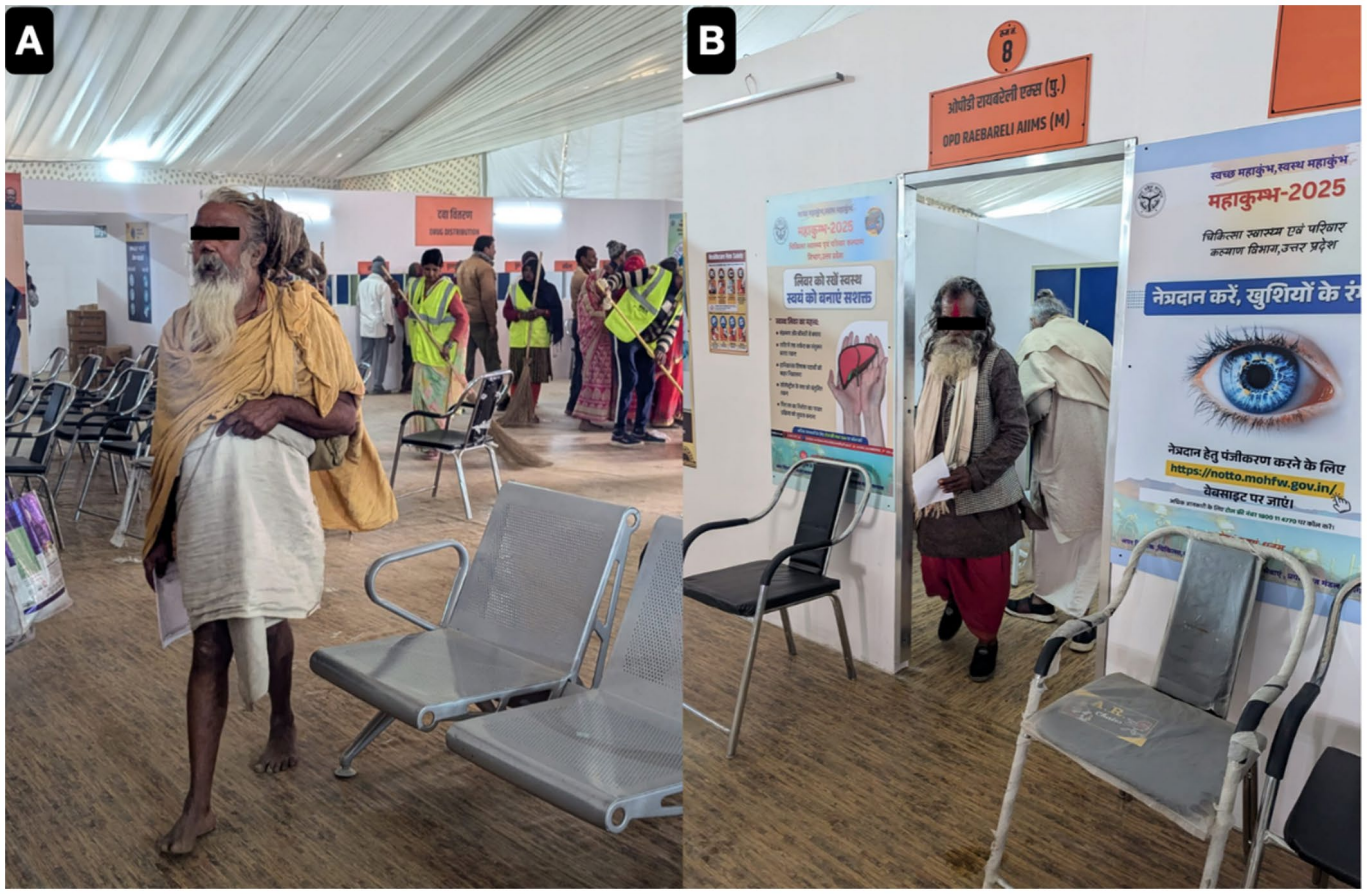
Representative photographs of our Out Patient Department Services **(A,B)** as serving the pilgrims from all around country.

The demographic profile revealed a significant male predominance (82.5%), with females accounting for only 17.5% (*p* < 0.001) of the patients. The observed male predominance likely reflects the gendered participation patterns in ritual bathing and the greater mobility of male pilgrims within the camp zones, consistent with prior Kumbh demographic reports. The majority of cases emerged from Uttar Pradesh (66.36%), followed by Bihar (17.73%), Madhya Pradesh (4.42%), and Maharashtra (3.92), while the northeastern and coastal states such as Sikkim (0.0052%), Manipur (0.0069%), Goa (0.012%), and Tripura (0.012%) (*p* < 0.001) showed the least representation. In our study, age-wise distribution depict the highest disease burden was observed in pilgrims aged 45–60 years (39.09%), followed by those above 60 (26.22%) and 30–45 years (21.07%). Notably, younger adults aged 18–30 years made up 10.45%, while children under 18 years accounted for only 3.034%, and infants represented just 0.13% of the total caseload (*p* < 0.001). The pattern of diseases observed revealed that communicable mainly Acute febrile illnesses formed a major portion of clinical presentations, fever contributes highest reporting ailments (30.62%), followed by symptoms associated with upper respiratory tract infections like cold and cough (23.85%) (*p* < 0.01), suggesting the circulation of seasonal viral or bacterial pathogens. Non-communicable conditions including body pain (12.25%), joint/back pain (5.42%), and asthma or breathlessness (0.21%) were documented. Other less frequent but notable diagnoses included epilepsy (0.2%), hypertension and chest pain (0.11%), ENT complaints, headache, and rare conditions such as cancer, depression, and diabetes; all representing less than 0.1% of cases (*p* < 0.05), in Figure 5 showing both OPD and IPD services as serving the pilgrims.

### Emergency department and ICU care: inpatient department data analyses

A total of 1,190 patients were admitted in Emergency during the Mahakumbh event at our institution. The cohort comprised 843 males (70.8%) and 316 females (26.6%), with gender unspecified in a few records (*p* < 0.001). The mean age of the patients was 50.9 years, ranging from 2 to 106 years. The average duration of stay in the hospital was 1 day, highlighting the short-term, high-throughput nature of care provided in this temporary setup. The common presenting complaints included (a) SOB: 447 cases, (b) Breathlessness: 39 cases, (c) Fever: 30 cases, (d) Loss of consciousness: 28 cases, (e) Chest pain: 52 cases, (f) SOB with fever: 20 cases, (g) Abdominal pain: 20 cases, (h) Seizures: 19 cases, (i) Diarrhoea: 17 cases (*p* < 0.05). These findings reflect the acute care demands and diverse clinical presentations managed efficiently in a mass gathering scenario. Other patients were admitted for generalised weakness and unconsciousness, giddiness, trauma, cut injuries, animal bites and exacerbation of COPD/asthma.

### Laboratory results: haematology and biochemical blood reports audit

At our AIIMS Mahakumbh facility, the addition of point-of-care diagnostics greatly improved emergency response. Timely interventions were directed by important assays, such as routine haematological and biochemical panels, CKMB, and ABG analysis. Notably, dual anti-platelet treatment and oxygen support were used to identify and treat 38 myocardial infarction cases (*p* < 0.01). In high-volume, resource-constrained settings, this military-inspired lab integration strategy proved essential for quick triage, stabilisation, and referral.

### Manpower and resource deployment: emphasis towards training and skill up-gradation

A total of seven teams were deployed on a rotational weekly basis to manage clinical and emergency care at the Mahakumbh Sub-Center Hospital. The first team, comprising four faculty members, two residents, and nine nursing officers, was posted prior to the hospital’s operational start with the primary objective of capacity building and skill enhancement. The Uttar Pradesh State Health Department provided infrastructure support, while AIIMS New Delhi and the Society of Acute Care, Trauma, and Emergency Medicine facilitated training and hand-holding for emergency preparedness.

#### BLS and emergency training programme

Training was conducted in two phases to ensure maximal preparedness:

- *Pre-deployment Phase at AIIMS, Raebareli:* Seven days of intensive training, including didactic lectures, hands-on practise, and case-based modules, were delivered to all medical, nursing, and support staff (hospital attendants, guards, technicians). Focus areas included Basic Life Support (BLS), CPR, airway management, cardiac arrest recognition, shock management, trauma stabilisation, emergency triage, and basic wound care. Participants practised using AEDs, manual defibrillators, bag-valve-mask ventilation devices, portable oxygen cylinders, suction apparatus, basic wound care kits, and CPR simulation mannequins in controlled scenarios replicating mass-gathering emergencies.
- *On-site Refresher Phase at Mahakumbh Hospital:* Conducted on the first day of deployment, this phase reinforced practical application of BLS and emergency protocols under high patient density, resource-limited ICU conditions, and rapid triage during peak ritual bathing days (*Amrit Snan*). Simulations included cardiac arrest management, respiratory emergencies, trauma stabilisation, and wound care.

#### Clinical team deployment post-training

- OPD Team: 1 faculty, 2 residents, 1 dresser, 1 guard (8 a.m.–8 p.m. shifts)
- ICU/Emergency Team: 2 faculty, 4 residents, 10 nursing officers, 4 attendants, 2 guards (24 × 7 coverage for 10 ICU beds)
- Laboratory Team: 1 faculty, 1 resident, 2 technicians, 1 attendant for biochemical and pathological investigations

Staff numbers were increased during expected patient surges on *Amrit Snan* days to ensure uninterrupted care.

#### Impact of training

This organised, equipment-supported BLS programme empowered individuals of all levels, including non-medical professionals, to actively engage in emergency response. It facilitated effective management of substantial patient volumes, acute cardiac events, respiratory emergencies, trauma, and wound care, even during peak crowd density. Conducting both pre-deployment and on-site training reinforced skills, ensured familiarity with protocols and equipment, and enhanced overall hospital readiness, reflecting a sustainable model for mass-gathering healthcare in resource-limited settings.

## Discussion

### History of the Mahakumbh Mela: background of the place where hospital was located

The Mahakumbh Mela, a sacred confluence that unfolds every 12 years, stands as the world’s largest spiritual gathering, deeply rooted in ancient Hindu mythology and cosmic symbolism. According to Hindu scriptures, this divine vessel emerged during the *Samudra Manthan* (churning of the cosmic ocean), containing *Amrita*, the nectar of immortality. The ritualistic immersion in the sacred rivers—notably the Ganges, Yamuna, and the mythical Saraswati—is the most defining practise of the Mahak*umbh*. During Mahakumbh, bathing in the holy waters is not only symbolic; it is also a Vedic method of spiritual and scientific purification. Additionally, it symbolises *“Tirtha,”* a journey to the holy, but it also purifies *karmic* weights and physical poisons. Today, its message of enlightenment and harmony attracts seekers from all over the world. The presence of international pilgrims underscores the scientific and philosophical depth of the event, fostering an intercultural exchange of wisdom and sacred traditions (2). During the sacred months of Mahakumbh 2025, from January 13 to February 26, Prayagraj welcomed an unprecedented influx of pilgrims, marking one of the largest mass gatherings in recorded history. According to the government’s statistics, about 15.36 million people have been placed under active medical surveillance, demonstrating the comprehensive safety measures in place. Over the 45-day span, the overall number of national and international pilgrims reached an astonishing 663 million (66.3 crore). Uttar Pradesh Government graciously managed this massive turnout, demonstrating a unique blend of spiritual hospitality and robust infrastructure, as Prayagraj stood at the heart of this holy confluence, welcoming every devotee into its sacred fold. The Uttar Pradesh Health Departments has planned 3–4 Sub-Centres and one central hospital to cater above pilgrims and our hospital was located at “*Sangam Marg*” the main lane for Sangam centrally situated with all the *Akharas*.

### Why 2025 Mahakumbh at Prayagraj was different?

**Figure 6:**
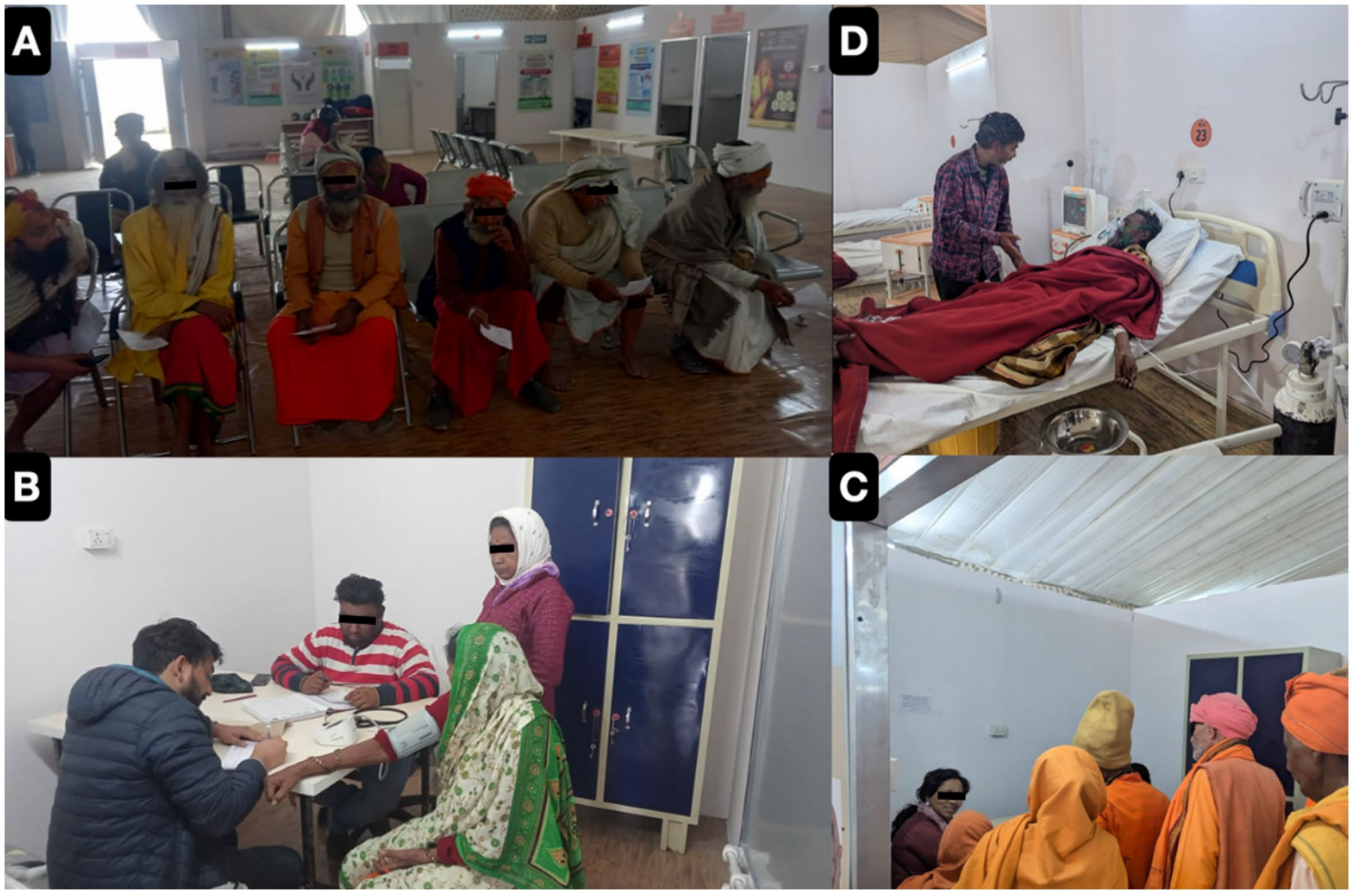
Representative photographs of our Out Patient Department Services **(A–C)** and Inpatient Department (IPD) **(D)** as serving the pilgrims from all around country.

Mass gatherings attract massive, varied populations from all over worldwide, increasing exposure to a variety of potential hazards and the risk of disease transmission (3). Globally, religious events draw millions of people, putting a strain on host healthcare systems and raising the possibility of infectious disease transmission, as shown in Figure 6 pilgrims and saints being admitted in our outreach camp hospital for breathing difficulties and exacerbation of respiratory diseases (e.g., COPD).

Strong public health surveillance, worldwide collaboration, and efficient planning are necessary to stop the spread of the disease and safeguard both local communities and pilgrims who are returning home (4–6). The Kumbh Mela, a major Hindu pilgrimage held every 4 years in four Indian cities; Prayagraj, Haridwar, Nashik, and Ujjain. The most recent, held in Haridwar from April 1 to 30, 2021, drew roughly 9.1 million pilgrims despite COVID-19 limitations. In contrast, the Mahakumbh Mela, which is recently held in Prayagraj from January 13 to February 26, 2025, witnessed an unprecedented turnout. By February 14, more than 500 million devotees had taken the holy dip, outnumbering the combined populations of the United States and Russia. This tremendous influx emphasises both the event’s profound spiritual significance and the monumental logistical efforts required to host this massive crowds (7–9).

**Figure 7:**
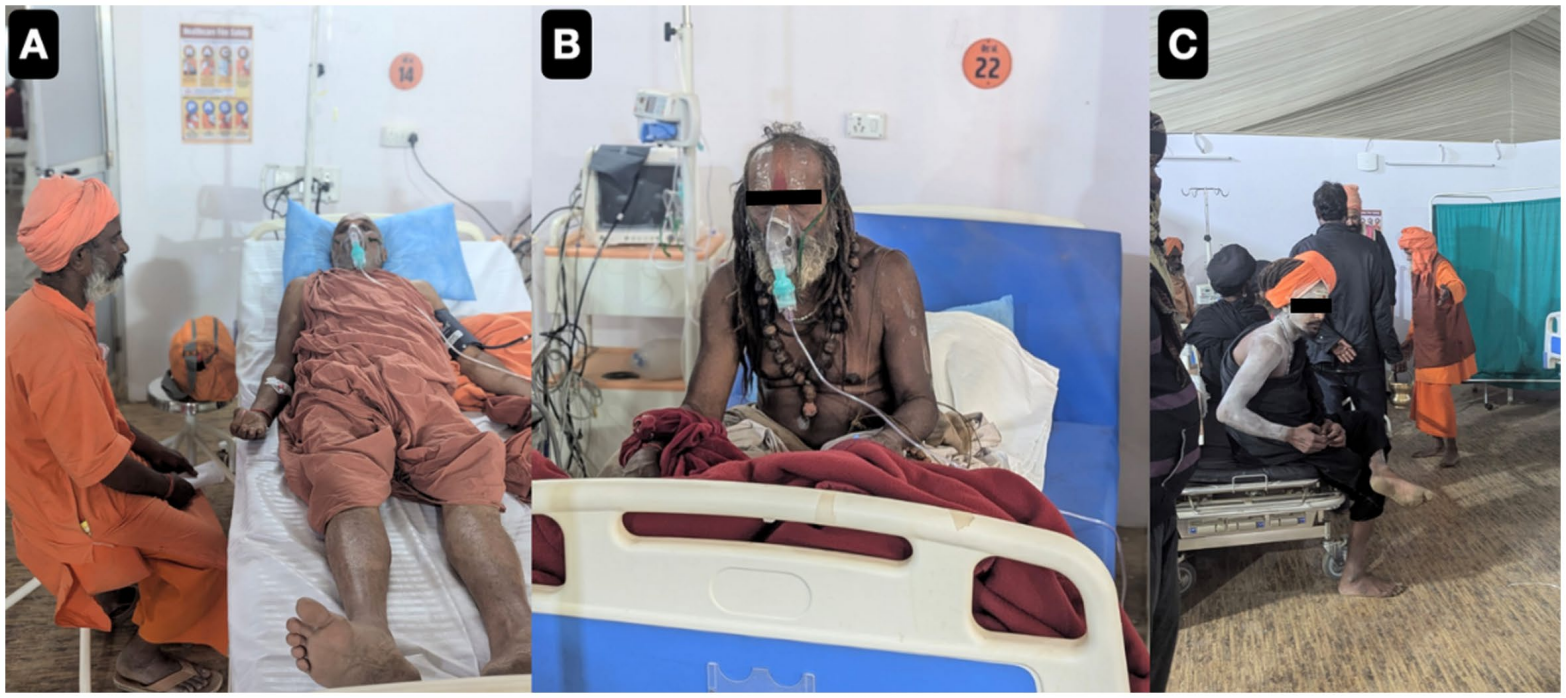
Representative photographs of massive mass gatherings of pilgrims and saints visited in our outreach camp hospital **(A–C)**.

The Mahakumbh Mela 2025 in Prayagraj marked an unprecedented milestone in the history of religious mass gatherings, with a cumulative footfall of approximately 663 million pilgrims by 26th February 2025, including 15.36 million on that single day alone. This massive turnout surpassed global gatherings like Hajj and major events, marking it as the largest human assembly. Such density presents serious public health risks, including limited mobility, environmental exposure, and increased infectious disease transmission (10–12). In anticipation of these challenges, a comprehensive healthcare preparedness plan was implemented, including the deployment of outreach medical health camps operational from 9th January to 27th February 2025, spanning the pre-Mahakumbh phase through the major ritual bathing days (*Amrit Snan*). During this surveillance window, 57,950 individuals were clinically evaluated and suspected to be suffering from various infectious diseases, as depicted in Figure 7, which highlights likely communicable diseases including respiratory distress and related ailments.

These findings reflect the immense strain placed on health systems and underscore the critical need for robust syndromic surveillance, rapid diagnostics, and adaptive public health strategies. This retrospective audit evaluates the epidemiological trends observed during the event, identifies gaps in surveillance efficacy, and informs future preparedness frameworks for mass gatherings of similar magnitude.

#### Epidemiological insights from Mahakumbh 2025: surveillance and disease trends

Mahakumbh 2025 in Prayagraj poses complicated public health challenges because to their unparalleled scale and density, with approximately 663 million pilgrims congregating in a small location. This environment considerably increases the risk of communicable disease transmission, including respiratory infections (Influenza, COVID-19, Tuberculosis), gastrointestinal illnesses (cholera, typhoid, hepatitis A and E), and vector-borne diseases (Dengue, Malaria, Chikungunya). The issue becomes worse by strained water, sanitation, and hygiene (WASH) infrastructure and prolonged exposure to harsh cold weather conditions, which create ideal conditions for epidemic amplification. Furthermore, the physical demands of pilgrimage add to the burden of non-communicable health emergencies, including cardiovascular incidents, diabetic crises, and neurological disorders. Trauma-related injuries, such as crush injuries, burns, and heat exhaustion, put additional strain on emergency health services, especially during ceremonial peaks like *Amrit Snan* (5, 13–15). Gastrointestinal symptoms such as constipation (22.02%) and Diarrhoea related complaints were also common, similar findings reported in the other studies (15).

#### Trends of the spread of infectious diseases during mass gatherings: Mahakumbh 2025

The public health hazards associated with massive crowds are vast and widespread (16). In the case of the Mahakumbh Mela, the unique ritual practise of *Snan* contributes to the health hazards associated with any mass gathering event. Mass bathing in the river has been shown to increase the bacterial load of the river’s water. Microbes carried by pilgrims on their skin, mucosa, and gastrointestinal tracts might contaminate the water. Despite sanitation initiatives, open defecation and urination continued during the 2013 and 2016 Kumbh Melas, contaminating the environment severely. During peak bathing days, river water bacterial load can increase by up to 130 times, according to studies, due to high population density, poor hygiene adherence, and waste disposal along riverbanks (17–20).

These locations become hotspots for faecal contamination, increasing the risk of enteric pathogen transmission from faeces to mouth. Similar sanitary problems were reported during Mahakumbh 2025, which attracted an unprecedented 663 million pilgrims, posing ongoing public health risks. The ritual practise of mass bathing (*Snans*; Holy bath) adds to these issues, emphasising the vital need for enhanced sanitation infrastructure, real-time water quality monitoring, and behavioural interventions to reduce the spread of waterborne infections (21, 22).

According to this retrospective surveillance from Mahakumbh 2025 data, gastrointestinal illnesses accounted for a significant portion of the disease burden, with 22.02% of patients reporting constipation and fewer but significant cases of diarrhoea, dysentery, and stomach pain accounting for approximately 0.1% of total diagnoses. These findings, together with the holy dip of mass bathing (*Snan*) and a previously recorded increase in bacterial contamination of river water, hint to a continued danger of waterborne infections like as diarrhoea, dysentery, and typhoid during the period of festivities. This emphasises how crucial it is to implement strong sanitation and water quality protocols at massive gatherings to avoid waterborne illnesses like cholera, typhoid, and diarrheal illnesses that have historically been connected to occasions like the Kumbh Mela (23). It is interesting to observe that there was a surge in cases of Acute febrile illness accounted for 30.62% (sudden onset of fever ≥38 °C/100.4°F lasting less than 2 weeks), of all diagnosed patients, according to the communicable disease surveillance conducted during Mahakumbh 2025. This suggests that undifferentiated febrile syndromes, which are likely to be associated with infectious aetiologies, are highly prevalent. While a lesser fraction showed COVID-19-like symptoms 0.05% and laboratory-flagged viral or bacterial infections 0.1%, cold and cough-related symptoms were noted in 23.85% of cases, indicating broad upper respiratory tract involvement. The increased danger of respiratory and systemic illness transmission in large crowds is shown by these data taken together (24).

### Temporal analysis of disease burden during Mahakumbh 2025

**Figure 8:**
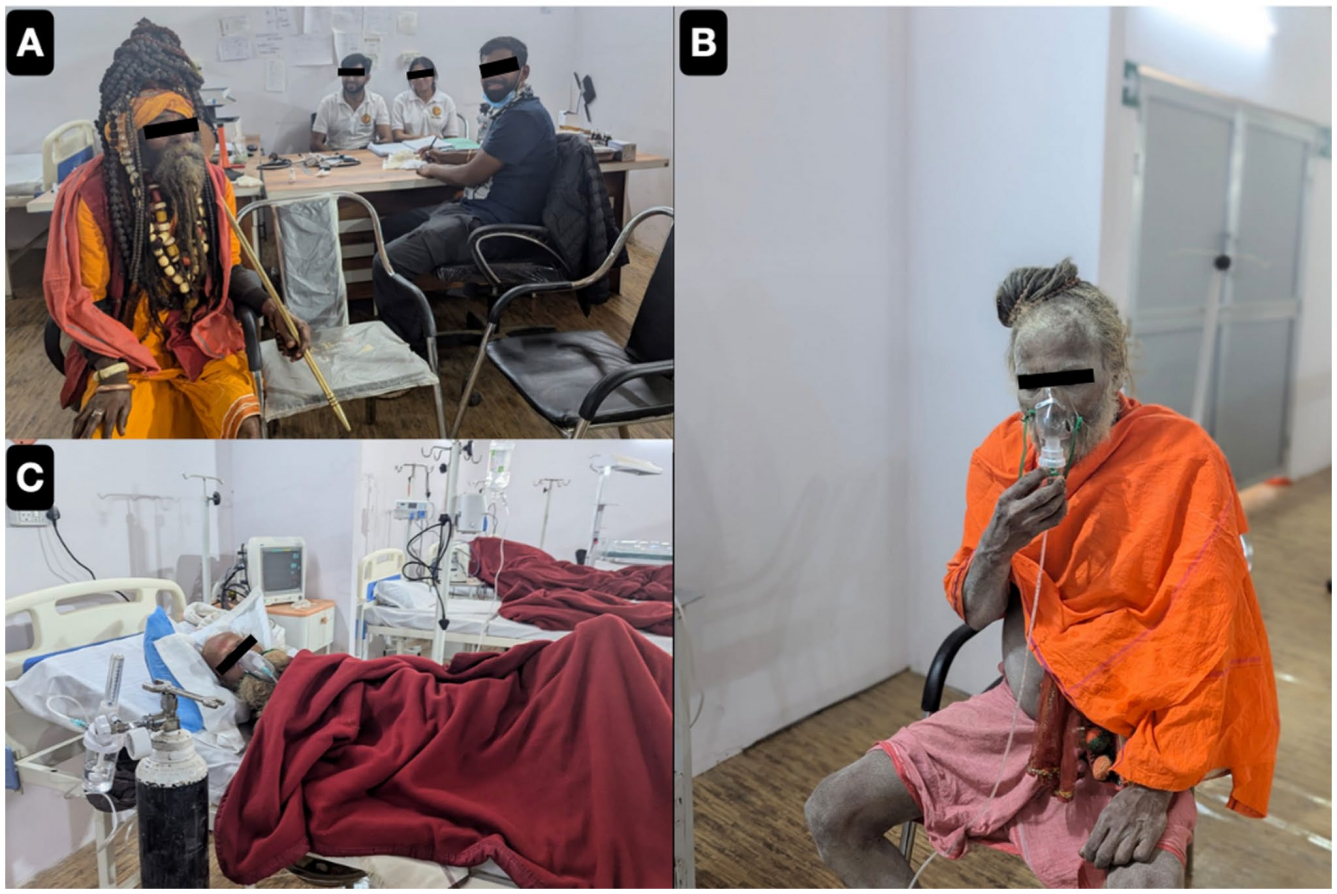
Representative photographs of pilgrims and saints being admitted in our outreach camp hospital for breathing difficulties and exacerbation of Chronic Obstructive Pulmonary Disease **(A–C)**.

Outpatient surveillance over the 45-day Mahakumbh 2025 indicated dynamic changes in morbidity patterns that were closely associated with the four *Amrit Snan* bathing rites and pilgrim demographics. In the initial phase (January 9–18, *Makar Sankranti*), consultations were largely attended by older adults pilgrims (>60 years; 32.82–39.11%), reflecting *sadhus* and saints traditional participation in early sacred bathing as shown in Figure 8, with some enduring the harsh winter with minimal or no clothing.

Cold/cough (35.01%) and fever (32.82%) were common, most likely owing to severe winter weather and minimal thermal protection, whereas constipation (28.87%) and bone/joint pain (39.11%) were associated with dietary changes and physical strain. Nearly 38% of individuals experienced other symptoms such as weakness, chest discomfort, and ENT difficulties, indicating the cumulative consequences of ageing and prolonged ritual participation. During *Mauni Amavasya* (January 19–28), morbidity was concentrated in older adults individuals, with fever (37.54%) and musculoskeletal discomfort (36.54%) prevailing, whereas middle-aged pilgrims (45–60 years, 30.51%) reported cough (28.82%) and constipation (26.92%) more frequently. Other symptoms, such as gastrointestinal discomfort and skin issues, increased by approximately 35%, indicating exhaustion and crowd-associated exposures.

Following *Mauni Amavasya*, the period from January 29 to February 7 recorded an upsurge in febrile illness among middle-aged (26.11%) and 30–45 year pilgrims (22.88%), as well as persistent constipation and respiratory complaints among older adults pilgrims, with nausea and headache emerging in 29% of cases—possibly due to the practise of fasting or ‘*Upvas’*, irregular meals, water contamination, and exhaustion. *Basant Panchami* (February 8–17) witnessed a significant shift in population, with middle-aged pilgrims accounting for over half of cases (49.05%) and reporting fever (49.05%), cough (39.54%), constipation (36.59%), and joint pain (43.89%). Nearly 47.91% reported other symptoms such as chest pain, asthma, and anxiety, which are symptoms associated with stress, pollution, and a prolonged stay.

**Figure 9:**
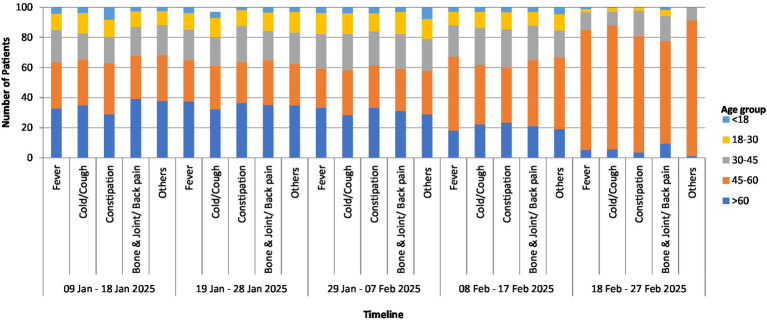
Temporal distribution of total hospital encounters and symptoms prevalence during Mahakumbh 2025.

By the end of the event (February 18–27, *Maha Shivratri*), middle-aged pilgrims dominated (79.83%), with respiratory illnesses particularly prevalent—cough (82.28%) and fever (79.83%), as well as constipation (77.21%) and bone pain (68.08%), while older adults individuals declined below 10% and younger groups under 5%. Notably, 90.14% of middle-aged pilgrims reported additional symptoms, including ENT difficulties, weakness, and psychological distress, highlighting the cumulative effect of prolonged crowding and ritual practises. These findings demonstrate how age structure, climate stresses, and ritual intensity influenced the temporal clustering of febrile, respiratory, musculoskeletal, and gastrointestinal illness during the Mahakumbh, comprehensive temporal observations are shown in the graphical depiction (Figure 9).

### Planning of material, manpower and logistics for Mahakumbh outreach camp hospital—skill up-gradation was the key

The management of critical care services during the Mahakumbh Mela, where 1,200 patients were successfully treated in the Emergency and ICU settings at Mahakumbh Hospital, presents a compelling example of mass-gathering medicine in action. This large-scale operation, involving 70 doctors, 70 nurses, and 40–50 support staff on a rotational basis, required not only medical preparedness but also meticulous planning across multiple domains including biomedical waste management, staff nutrition, laundry services, transportation, and cold-weather operations.

Our experience highlights the vital significance of all-encompassing health plans for large-scale events, like the Hajj (23–25). To reduce the danger of infectious diseases, heatstroke, and crowd-related illnesses, coordinated multi-agency activities, mobile clinics, vaccination campaigns, and effective outbreak control are crucial. The Mahakumbh Hospital’s operational success emphasises the importance of strategic personnel rotation, efficient transport logistics, and resilience in challenging circumstances mirroring Hajj models ensuring sustained clinical performance, reduced staff fatigue, and continuous healthcare delivery during high-demand mass gatherings (23, 25). A critical aspect often under appreciated in such deployments is biomedical waste management. In a setting where infectious risks are high and disposal systems are challenged by temporary infrastructure, the implementation of strict protocols was pivotal. WHO guidelines on healthcare waste management in emergencies emphasise the importance of segregation, transport, and final disposal using incineration or deep burial in resource-limited settings (26).

Moreover, linen and laundry services, especially in the context of ICU-level infection control, posed a considerable challenge due to the ambient cold weather. Special arrangements for heating and rapid turnaround of sanitised linen were necessary to maintain hygiene and comfort. Experiences from temporary health camps during the *Amarnath Yatra* in Kashmir, another religious pilgrimage with cold weather, also highlight the significance of pre-sterilised disposable linens and modular laundry units (27).

### Lessons learnt from emergency patient management in Mahakumbh Prayagraj 2025

The management of 1,190 patients during the Mahakumbh event highlights the scale and complexity of healthcare delivery in mass religious gatherings. A significant male predominance (70.8%) and an average patient age of 50.9 years are consistent with trends observed in previous Kumbh Melas and other religious events, where middle-aged and older adults men comprise a large proportion of attendees (28).

**Figure 10:**
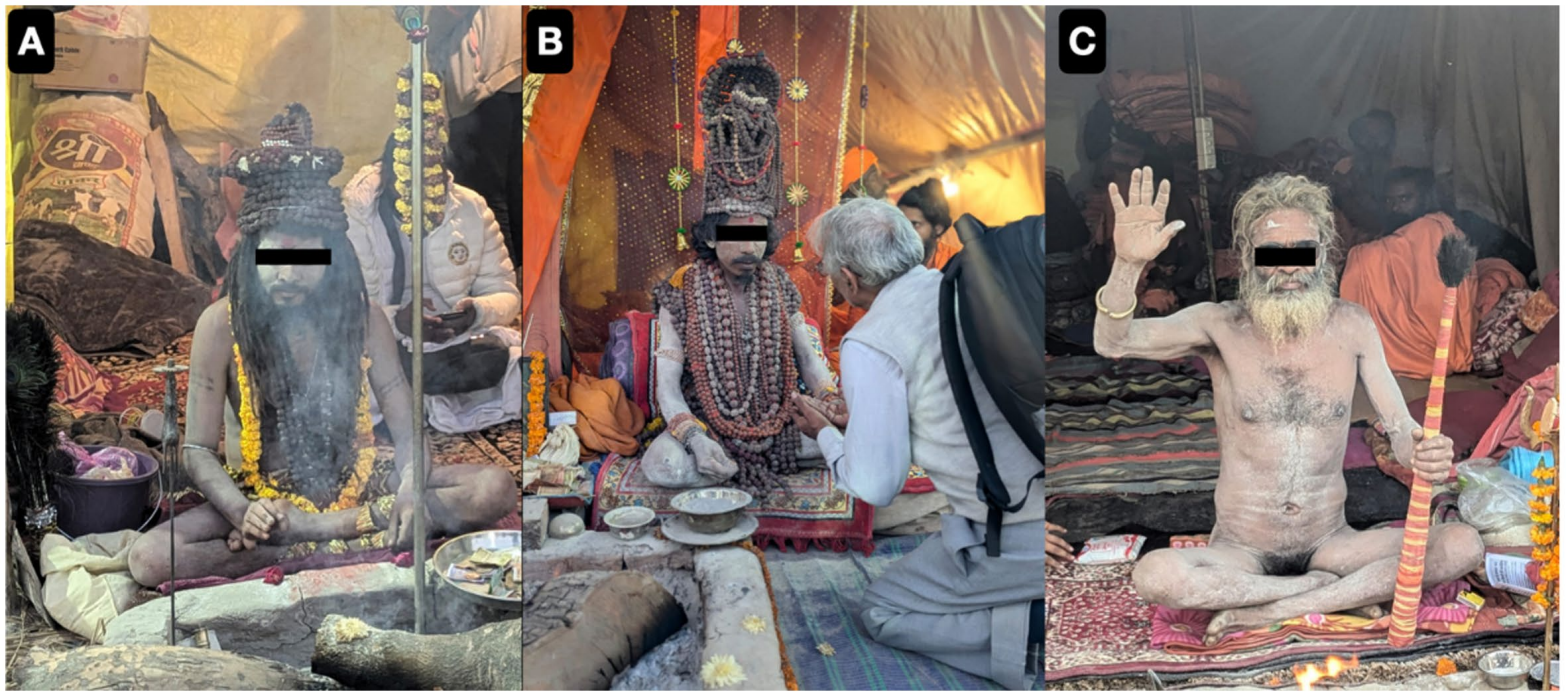
Representative photographs of saints **(A–C)** in Mahakumbh 2025.

**Figure 11:**
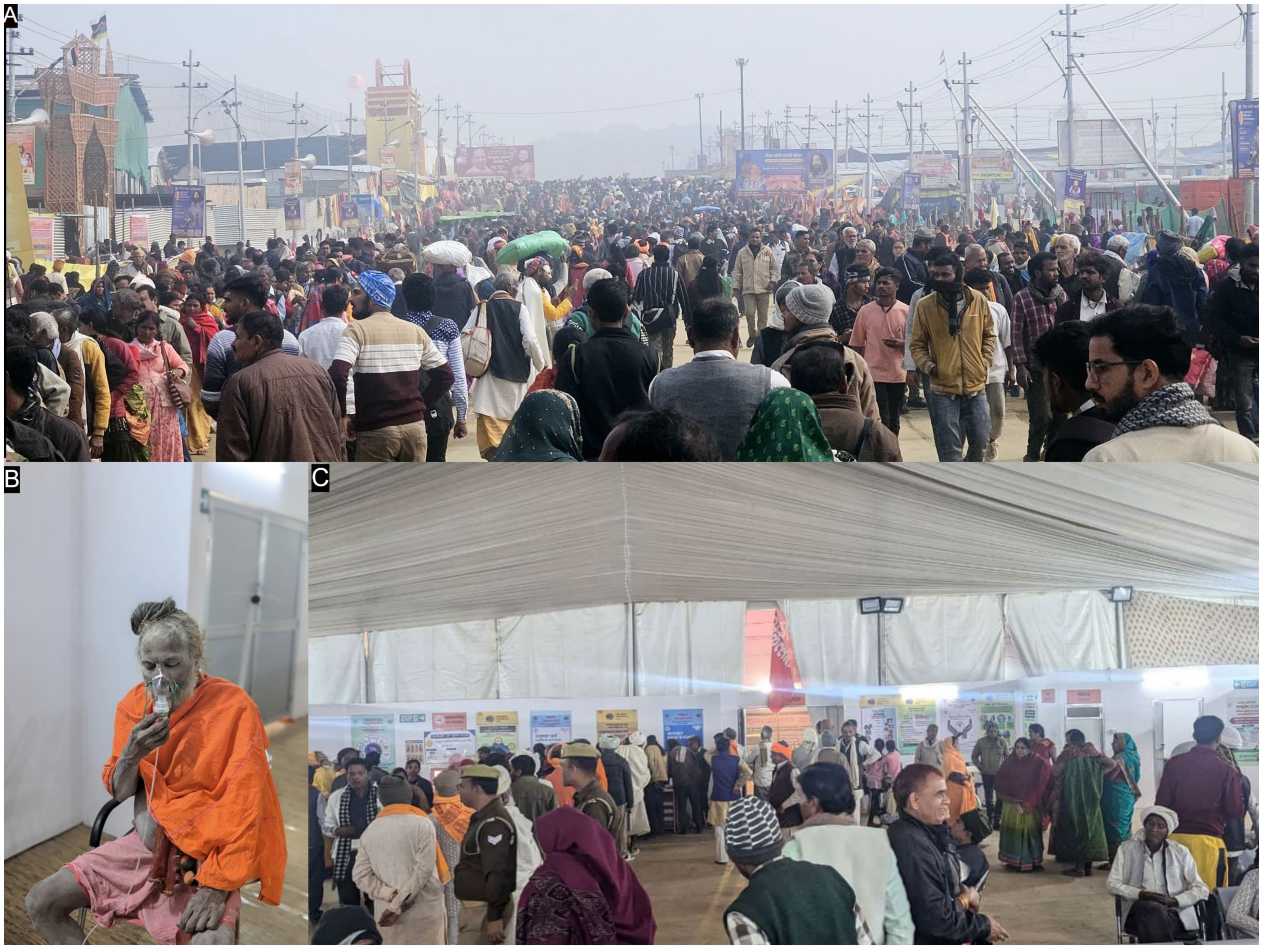
Representative photographs of massive mass gatherings of pilgrims and saints visited in our outreach camp hospital **(A, B** and **C)**.

Acute presentations dominated the clinical profile, with SOB, fever, fainting, and gastrointestinal symptoms being most frequent. These findings mirror the disease patterns documented during the Prayagraj Kumbh Mela in 2019, where 95% of illnesses were communicable, particularly acute respiratory infections and fevers (28, 29). The prevalence of respiratory symptoms, especially shortness of breath (SOB), may be linked to overcrowding, dust exposure, and pre-existing cardiorespiratory conditions among pilgrims as demonstrated by Figure 10, capturing congested mass gatherings with heavy dust exposure (Figure 11).

The average hospital stay of 1 day indicates a high-throughput, short-stay care model tailored for temporary health setups. Similar models have been used effectively during the Hajj pilgrimage, where health systems are designed to prioritise rapid triage, stabilisation, and discharge (29). Such systems are essential in preventing congestion and enabling the treatment of large volumes of patients with limited resources. Notably, the “brought dead” category (*n* = 41) underscores the importance of emergency medical preparedness. Although major outbreaks or disasters were averted, the potential risks remain substantial, as illustrated by incidents such as the fatal stampede at a Hindu gathering in Uttar Pradesh in 2024 (30). This emphasises the need for robust crowd management protocols and rapid emergency response capabilities.

Previous research from the Hajj and Kumbh Melas has also documented challenges such as the transmission of antibiotic-resistant infections and the need for vigilant infection control (31). Fortunately, no such events were reported in this dataset, reflecting effective sanitation, surveillance, and rapid response strategies. Disease surveillance systems, like those employed during the 2019 Kumbh Mela, have proven crucial for early outbreak detection and response (25, 31).

The high incidence of asthma and COPD exacerbations at Mahakumbh Hospital emphasises the impact of the Ganga riverbank’s sandy terrain, where mass pilgrim movement generated particulate matter (PM), exacerbating respiratory distress, particularly among vulnerable populations with pre-existing pulmonary conditions. Similarly with the 2013 Kumbh Mela, respiratory illnesses and discomfort were the most prevalent medical conditions during Mahakumbh 2025 (32). Elevated PM10 and PM2.5 levels during events along with crowd density, contributed substantially to increased respiratory symptoms (33). Although our dataset did not evaluate direct air-quality indicators, subsequent CPCB monitoring reports showed moderate to high PM2.5 levels during peak bathing days, corroborating the reported respiratory pattern. Future surveillance frameworks that incorporate real-time environmental data could strengthen causal inferences between particulate exposure and clinical outcomes in mass-gathering events.

Evidence from Hajj studies confirms that excessive particulate matter exposure and density have a major negative impact on respiratory health at large religious gatherings (34, 35).

Implementing dust management, providing masks, improving respiratory care, and issuing targeted health advisories can considerably lower respiratory risks during future large-scale gatherings (35, 36). Furthermore, real-time air quality monitoring and public health awareness campaigns can play a crucial role in reducing exposure and prompting early medical attention (37).

Early identification of myocardial infarctions in 38 patients was made possible by the use of military-grade diagnostic methods like CK-MB and ABG testing. Rapid cardiac triage is helpful in mass-gathering situations, as evidenced by the clinical stabilisation and safe referral for PCI that followed the prompt administration of anti-platelet medication and oxygen support (38–40).

### Future directions and institutional strategies

The Prayagraj Mahakumbh 2025 experience emphasises the vital role of incorporating structured, military-grade diagnostic and treatment protocols into civilian healthcare preparedness for large-scale public gatherings. Drawing from our institutional operations, this includes pre-event simulations, defined command hierarchy, and integrated digital reporting mechanisms. Establishing round-the-clock laboratory services, such as ABG and CK-MB testing, significantly enhanced the hospital’s ability to treat emergency situations efficiently. Military medical standards can assist in standardising emergency response processes and enhancing multidisciplinary team readiness through structured training and rapid-deployment modules. Prioritising well-equipped temporary medical facilities, AI-driven operational dashboards, interoperable communication systems, and a Centralised Incident Command System (ICS) can improve the coordination of health, security, logistics, and emergency operations. Furthermore, continuous capacity building—through refresher BLS/ACLS training, evidence-based triage protocols, and real-time performance audits—can ensure sustained preparedness during prolonged mass gatherings.

Importantly, this experience generated several new operational insights beyond previous Kumbh or Hajj literature. The integration of real-time digital surveillance dashboards with on-ground triage data shortened diagnostic turnaround and improved patient routing. Modular skill-upgradation sessions before deployment created a replicable “training cascade” model for temporary health facilities. The hybrid civil–military command structure and interoperable communication channels established during Mahakumbh offer a practical template for the National Disaster Management Authority (NDMA) and align with WHO’s Mass Gathering Medicine framework. Incorporating responder health monitoring aligned with the ERHMS framework further ensured staff well-being, resilience, and continuity of operations during prolonged deployment. Together, these learnings provide a scalable, evidence-informed model for national and international mass-gathering preparedness. Although this study’s single-centre retrospective design may limit broader generalisability, its strengths lie in comprehensive real-time surveillance, integrated diagnostics, and coordinated operations. Collectively, these elements position the AIIMS Mahakumbh model as a replicable and pioneering institutional framework for evidence-based mass-gathering health preparedness in India and similar large-scale events globally.

## Conclusion

The 2025 Mahakumbh experience highlights that structured preparedness, active surveillance, and coordinated emergency response can ensure efficient healthcare delivery even in high-density, resource-constrained environments. The observed surge in respiratory (asthma, COPD) and cardiac (acute MI) events reflects the importance of environmental health monitoring and rapid diagnostics. Strengthening triage systems, AI-driven surveillance, telemedicine integration, and interoperable data platforms can further enhance resilience and real-time decision-making for future mass-gathering health preparedness.

## Data Availability

The datasets presented in this article are not readily available. Requests to access the datasets should be directed to dr.suyashsingh@gmail.com.
